# Synthesis and Evaluation of a ^18^F-Labeled Ligand for PET Imaging of Colony-Stimulating Factor 1 Receptor

**DOI:** 10.3390/ph15030276

**Published:** 2022-02-23

**Authors:** Hyeokjin Lee, Ji-Hun Park, Hyunjung Kim, Sang-keun Woo, Joon Young Choi, Kyung-Han Lee, Yearn Seong Choe

**Affiliations:** 1 Department of Nuclear Medicine, Samsung Medical Center, Sungkyunkwan University School of Medicine, Seoul 06351, Korea; inje081406@naver.com (H.L.); rpdlsqhd@naver.com (J.-H.P.); hkim597@gmail.com (H.K.); jynm.choi@samsung.com (J.Y.C.); khnm.lee@samsung.com (K.-H.L.); 2 Division of RI-Convergence Research, Korea Institute of Radiological and Medical Sciences, Seoul 01812, Korea; skwoo@kirams.re.kr; 3 Department of Health Sciences and Technology, SAIHST, Sungkyunkwan University, Seoul 06355, Korea

**Keywords:** colony-stimulating factor 1 receptor, neuroinflammation, microglia, PET, ^18^F, CPPC

## Abstract

Neuroinflammation involves activation of glial cells in the brain, and activated microglia play a particularly important role in neurodegenerative diseases such as Alzheimer’s disease (AD). In this study, we developed 5-cyano-*N*-(4-(4-(2-[^18^F]fluoroethyl)piperazin-1-yl)-2-(piperidin-1-yl)phenyl)furan-2-carboxamide ([^18^F]**1**) for PET imaging of colony-stimulating factor 1 receptor (CSF1R), an emerging target for neuroinflammation imaging. Non-radioactive ligand **1** exhibited binding affinity comparable to that of a known CSF1R inhibitor, 5-cyano-*N*-(4-(4-methylpiperazin-1-yl)-2-(piperidin-1-yl)phenyl)furan-2-carboxamide (CPPC). Therefore, we synthesized radioligand [^18^F]**1** by radiofluorination of chlorine-substituted precursor **7** in 13–15% decay-corrected radiochemical yield. Dynamic PET/CT images showed higher uptake in the lipopolysaccharide (LPS)-treated mouse brain than in control mouse brain. Ex vivo biodistribution study conducted at 45 min after radioligand injection showed that the brain uptake in LPS mice increased by 78% compared to that of control mice and was inhibited by 22% in LPS mice pretreated with CPPC, indicating specificity of [^18^F]**1** for CSF1R. A metabolism study demonstrated that the radioligand underwent little metabolism in the mouse brain. Taken together, these results suggest that [^18^F]**1** may hold promise as a radioligand for CSF1R imaging.

## 1. Introduction

Neuroinflammation is attributed to activation of glial cells. In the brain, glial cells are involved in maintaining homeostasis and protecting the brain from infection or damage [1,2]. Unlike the other glial cells, microglia, which make up 0.5–16.6% of all cells in the human brain, are specialized resident macrophage cells in the central nervous system (CNS) [3]. As the innate immune cells of the CNS, microglia serve as key mediators of the immune system in the brain [4]. Therefore, activation of the immune system in response to inflammatory stimuli is considered to play an important role in the initiation of neuroinflammation [5,6]. Moreover, neuroinflammation has long been associated with neurodegenerative diseases [7]. Substantial evidence has shown that neuroinflammation characterized by activated microglia contributes to the onset and progression of neurodegenerative diseases, such as Alzheimer’s disease (AD) [8,9]. Therefore, early detection of neuroinflammation can be beneficial for the diagnosis and monitoring of neurodegenerative diseases.

There has been increased interest in potential molecular targets for PET studies of neuroinflammation, including translocator protein-18 kDa (TSPO), purinergic receptor P2X_7_, cyclooxygenase, cannabinoid-2 receptor, imidazoline-2 binding sites, and reactive oxygen species [9,10,11]. Of these, TSPO has been studied most intensively, and many radioligands for imaging of TSPO have been developed and used clinically. In conditions of neuroinflammation, TSPO expression is changed; it is upregulated in AD [12]. However, a single nucleotide polymorphism in the TSPO gene causes changes in the binding affinity of radioligands, and there can be large variations in PET data among individuals, which could impose some limitations on the clinical use of TSPO radioligands [13,14,15]. Colony-stimulating factor 1 receptor (CSF1R), a class III family of receptor tyrosine kinase, is one of the targets emerging for neuroinflammation imaging [9,13,16]. CSF1R, which is expressed mostly on the cell surface of microglia in the brain, has two homodimeric ligands, CSF1 and interleukin-34 (IL-34) [17,18]. Binding either of these ligands to CSF1R induces activation of CSF1R in a similar fashion [19]. CSF1R regulates microglial function, including maintenance, activation, proliferation, and self-renewal of microglia, which are associated with neuroinflammation [20]. Moreover, CSF1R is known to play a key role in neurodegenerative diseases, including AD [21]; expression of CSF1R was enhanced in microglia near β–amyloid plaques in transgenic mice and was upregulated in postmortem brain tissues of AD patients [22,23].

**Figure 1:**
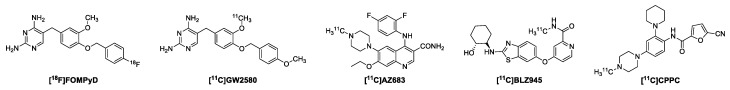
Known CSF1R PET radioligands.

Radioligands based on CSF1R inhibitors have been developed for PET studies of neuroinflammation (Figure 1), such as 5-(4-((4-[^18^F]fluorobenzyl)oxy)-3-methoxybenzyl)pyrimidine-2,4-diamine ([^18^F]FOMPyD) [24,25], 5-(3-[^11^C]methoxy-4-((4-methoxybenzyl)oxy)benzyl)pyrimidine-2,4-diamine ([^11^C]GW2580) [26], 4-(2,4-difluoroanilino)-7-ethoxy-6-(4-[^11^C]methylpiperazin-1-yl)quinoline-3-carboxamide ([^11^C]AZ683) [27], 4-((2-(((1*S*,2*R*)-2-hydroxycyclohexyl)amino)benzo[*d*]thiazol-6-yl)oxy)-*N*-[^11^C]methylpicolinamide ([^11^C]BLZ945) [28], and 5-cyano-*N*-(4-(4-[^11^C]methylpiperazin-1-yl)-2-(piperidin-1-yl)phenyl)furan-2-carboxamide ([^11^C]CPPC)) [18,29]. FOMPyD, a derivative of GW2580 (IC_50_ = 10 nM for CSF1R), was reported as a dual type-II tropomyosin receptor kinase (TrK)/CSF1R inhibitor (IC_50_ = 169 nM for CSF1R) [24,25]. Its ^18^F-labeled form, [^18^F]FOMPyD showed high non-specific binding in autoradiography of rat and human brain tissues, and the binding was not blocked with a known CSF1R inhibitor (BLZ945) [25]. Recently developed [^11^C]GW2580 demonstrated higher sensitivity than [^11^C]CPPC in PET imaging of acute and chronic inflammation mouse models [26]. It also displayed moderate-to-high brain uptake in a rhesus monkey, which was blocked by 30% with GW2580 [26]. AZ683 exhibited high and selective binding affinity for CSF1R (IC_50_ = 6 nM); however, its ^11^C-labeled radioligand had very low brain uptake in a rat and a non-human primate, and instead, it had high uptake in the pituitary and thyroid glands that is more likely due to its non-specific binding [27]. BLZ945 also displayed high binding affinity for CSF1R (IC_50_ = 6.9 nM), but [^11^C]BLZ945 was found to be a substrate for efflux transporters. Moreover, PET imaging and in vitro autoradiography of [^11^C]BLZ945 revealed high non-specific binding in mouse brain [28]. [^11^C]CPPC was developed based on a potent CSF1R inhibitor, CPPC (IC_50_ = 0.78 nM) [30] and showed specific brain uptakes in the lipopolysaccharide (LPS) mouse model and a non-human primate, compared with their control counterparts [18]. It also showed high and specific uptakes in a transgenic AD mouse model and an experimental autoimmune encephalopathy mouse model of multiple sclerosis, and on autoradiography of postmortem AD brain tissue sections [18]. 

Although [^11^C]CPPC was shown to have favorable biological properties for CSF1R imaging, it has a short half-life (20.4 min) that limits its multi-dose synthesis. In this study, therefore, we synthesized a ^18^F-labeled derivative of CPPC with a longer half-life (109.8 min), 5-cyano-*N*-(4-(4-(2-[^18^F]fluoroethyl)piperazin-1-yl)-2-(piperidin-1-yl)phenyl)furan-2-carboxamide ([^18^F]**1**), and evaluated it for CSF1R imaging.

## 2. Results and Discussion

### 2.1. Chemical Synthesis

**Scheme 1:**
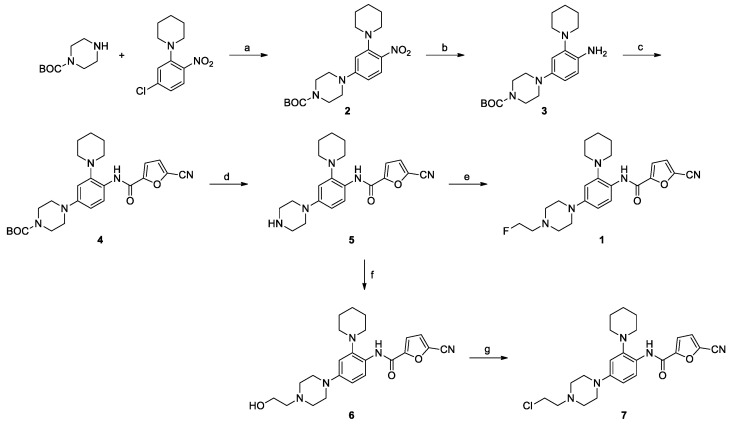
Reagents and conditions: (**a**) K_2_CO_3_, DMSO, 110 °C, 16 h; (**b**) SnCl_2_·2H_2_O, ethanol, 70 °C, 0.5 h; (**c**) 5-cyanofuran-2-carboxylic acid, HATU, DIPEA, DMF, rt, 16 h; (**d**) CF_3_COOH, CH_2_Cl_2_, rt, 16 h; (**e**) 1-fluoro-2-iodoethane, K_2_CO_3_, DMF, 65 °C, 6 h; (**f**) 1-bromo-2-hydroxyethane, K_2_CO_3_, DMF, rt, 16 h; (**g**) TsCl, Et_3_N, DMAP, CH_2_Cl_2_, rt, 16 h.

Non-radioactive ligand **1** was synthesized from 5-cyano-*N*-(4-(piperazin-1-yl)-2-(piperidin-1-yl)phenyl)furan-2-carboxamide (**5**) and 1-fluoro-2-iodoethane in the presence of K_2_CO_3_ (Scheme 1). Compound **5** was synthesized according to the literature with minor modifications [18]. Compound **2** was synthesized from 1-(5-chloro-2-nitrophenyl)piperidine and *tert*-butyl piperazine-1-carboxylate in the presence of K_2_CO_3_. The nitro group of compound **2** was easily converted to an amino group using SnCl_2_. The resulting compound **3** was reacted with 5-cyanofuran-2-carboxylic acid in the presence of 1-[bis(dimethylamino)methylene]-1*H*-1,2,3-triazolo[4,5-b]pyridinium 3-oxide hexafluorophosphate (HATU) and *N*,*N*-diisopropylethylamine (DIPEA) to give compound **4**. Removal of the *tert*-butoxycarbonyl (BOC) group from **4** under acidic conditions gave compound **5**.

For the fluoroethylation reaction on the piperazinyl NH group of **5**, we initially attempted to use 2-fluoroethyl tosylate as the fluoroethylating agent, but no desired product was produced. Alternatively, we tried to synthesize the tosylate precursor from **5** and ethylene di(*p*-tosylate) for subsequent fluorination, but this reaction did not give the tosylate product. Therefore, we synthesized compound **6** and tried to tosylate or mesylate its hydroxyl group. However, the reaction gave the chlorine-substituted compound **7** instead of the tosylate or mesylate precursor. This can be explained by the formation of unstable aziridinium salt, which undergoes ring-opening by attack of the chloride ion [31,32]. The use of *p*-toluenesulfonic anhydride as a tosylating agent also failed to yield the tosylate precursor. Therefore, we used 2-chloroethyl compound **7**, a by-product of the reaction of **6** with tosyl chloride, as the precursor for synthesis of ^18^F-labeled product, [^18^F]**1** (Scheme 1).

### 2.2. Radiochemical Synthesis of *[^18^F]**1***

**Scheme 2:**
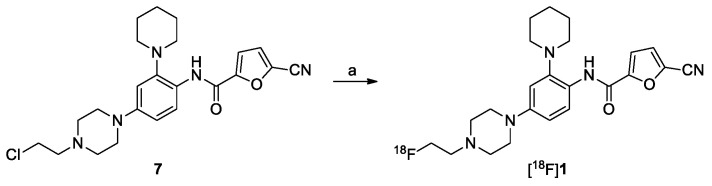
Reagents and conditions: (**a**) K[^18^F]F-K_2.2.2_, DMSO, 120 °C, 10 min.

[^18^F]Fluoroethylation reactions of amino group-containing compounds are well reported; generally used [^18^F]fluoroethylating agents include 2-[^18^F]fluoroethyl bromide, 2-[^18^F]fluoroethyl triflate, and 2-[^18^F]fluoroethyl tosylate. Among these agents, 2-[^18^F]fluoroethyl tosylate has advantages because of its easy preparation, low volatility, and high stability [33]. Hence, we attempted synthesis of [^18^F]**1** via *N*-[^18^F]fluoroethylation of **5** using 2-[^18^F]fluoroethyl tosylate. However, an unidentified polar radiolabeled by-product was obtained in higher yield than the desired product based on radio-thin layer chromatography (TLC) analysis. Addition of alkali iodide to the reaction of **5** with 2-[^18^F]fluoroethyl tosylate did not improve the product yield [34]. Previously, nucleophilic substitution reaction of the chlorine atom of 4-(2-chloroethyl)piperazinyl compounds with ^18^F has been reported [31,32]. Therefore, we conducted a one-step radiofluorination of chlorine-substituted precursor **7** at 120 °C for 10 min. The progress of the reaction was monitored by radio-TLC using a 20:1 mixture of CH_2_Cl_2_ and CH_3_OH as the mobile phase, and the radiochemical conversion in the radiofluorination step was 30–35%. At the end of synthesis, the reaction mixture was extracted with EtOAc, and the organic phase was dried and purified by HPLC. This radiochemical synthesis procedure may be improved by diluting the reaction mixture with water prior to HPLC purification to avoid the extraction step and by using solid-phase extraction cartridges for formulation of the product [35]. The total synthesis time was 80–85 min. [^18^F]**1** was obtained in an overall 13–15% decay-corrected radiochemical yield (8–8.5% non-decay corrected yield) and radiochemical purity higher than 99% with a molar activity of 32–38 GBq/μmol (n = 3) (Scheme 2). In this study, a low dose of [^18^F]fluoride ion was used for the manual synthesis of [^18^F]**1**. Thus, the molar activity of the radioligand would increase greatly if a higher dose of [^18^F]fluoride ion is used.

### 2.3. In Vitro Studies

The binding affinity of ligand **1** for CSF1R was measured using a fluorescence resonance energy transfer (FRET)-based method. The binding affinity of CPPC, a known CSF1R inhibitor, was measured at the same time for comparison. The IC_50_ value of CPPC was 1.56 ± 0.08 nM (Table 1, Figure S1), while its reported IC_50_ value was 0.78 nM when measured using a fluorescence polarization competition method [30]. As shown in Table 1 and Figure S1, the IC_50_ value of **1** was found to be 3.42 ± 0.33 nM, indicating that **1** also has high binding affinity for CSF1R. This result suggests that 2-fluoroethyl substituent at the piperazine moiety of the ligand is tolerated for binding to CSF1R, although it is less tolerated than a methyl substituent as in CPPC. Therefore, we synthesized radioligand [^18^F]**1** for in vitro and in vivo evaluation.

The octanol-water partition coefficient of [^18^F]**1** was measured, and its log *p* value was 2.30 ± 0.01, suggesting favorable brain permeability of the radioligand.

**Figure 2:**
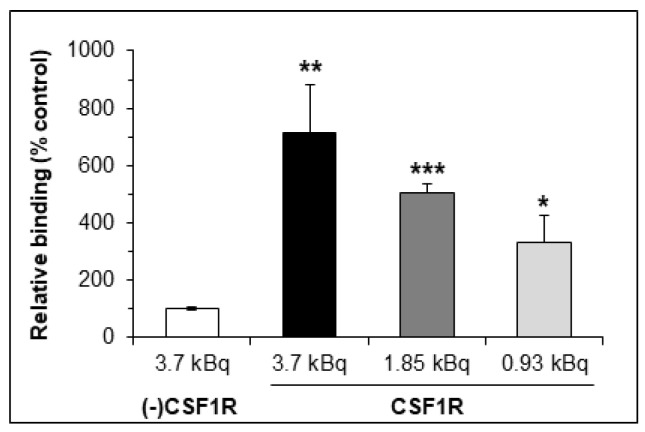
In vitro binding of [^18^F]**1** to CSF1R. Binding levels are expressed relative to a value of 100 % for control (without CSF1R). Data represent mean ± S.D. (n = 3). * *p* < 0.05, ** *p* < 0.01, *** *p* < 0.001.

A binding study of radioligand [^18^F]**1** for CSF1R was also performed using well plates coated with CSF1R. As shown in Figure 2, the radioligand bound to CSF1R-coated wells was 7.14-times higher than the radioligand bound to non-coated wells (control). Moreover, the binding levels of the radioligand to CSF1R decreased with lower doses of the radioligand: 7.14-fold with 3.7 kBq, 5.07-fold with 1.85 kBq, and 3.31-fold with 0.93 kBq, relative to the value of control (** *p* < 0.01, *** *p* < 0.001, and * *p* < 0.05). This result suggested that [^18^F]**1** binds to CSF1R in vitro.

### 2.4. In Vivo Studies

**Figure 3:**
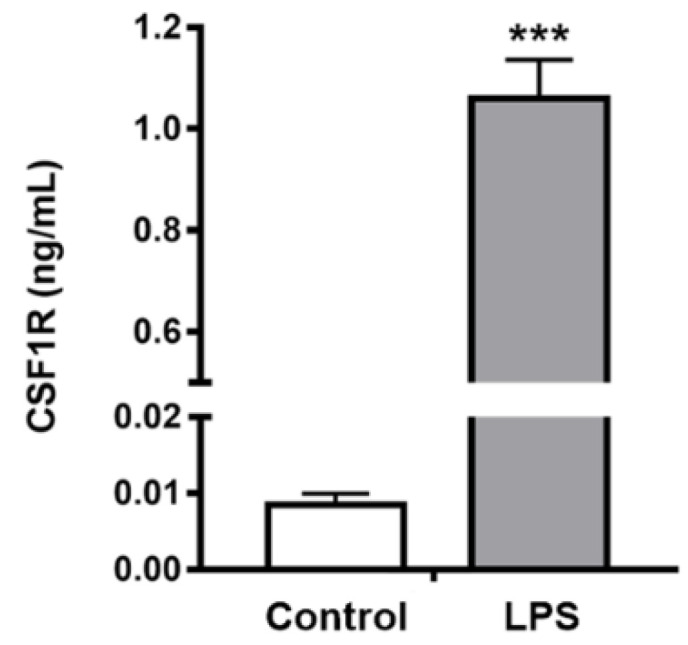
CSF1R expression levels in brain tissues from control (white) and LPS mice (grey). Brain tissues were collected on the third day after i.p. injection of saline (control) or LPS into mice. Data represent the mean ± S.D. (n = 3). *** *p* < 0.001.

An LPS mouse model of neuroinflammation can be prepared by injecting LPS into mice either intracranially or intraperitoneally (i.p.) [18,36]. Horti et al. obtained comparable results from the two mouse models in their studies [18]. In our studies, therefore, we produced the LPS mouse model by injecting LPS (10 mg/kg) i.p. into mice [18]. The expression levels of CSF1R in mouse brain tissues were measured using an enzyme-linked immunosorbent assay (ELISA) on the third day after i.p. injection of LPS or saline into mice (Figure 3). The CSF1R levels were significantly higher in the brains of LPS-treated mice than in the brains of control mice, by a factor of 124 (*** *p* < 0.001). Therefore, we conducted in vivo studies on the third day after i.p. administration of LPS into mice.

**Figure 4:**
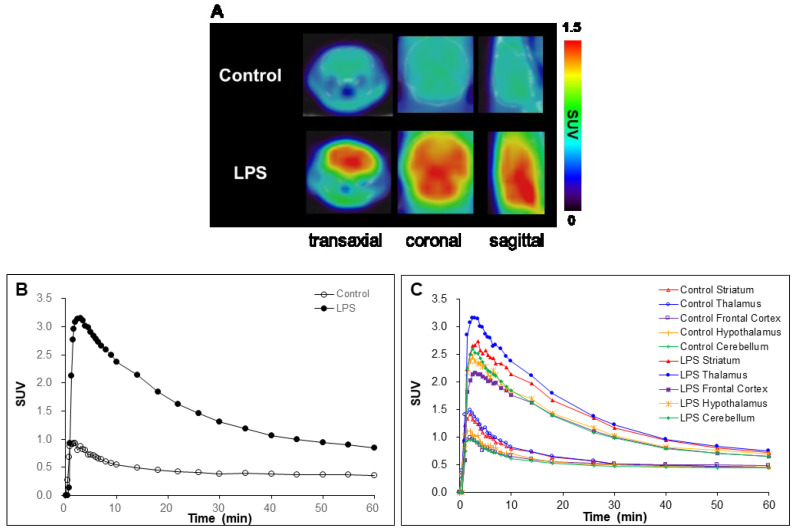
(**A**) Dynamic PET images of control and LPS mice acquired for 60 min after injection of [^18^F]**1**; (**B**) whole brain TACs of [^18^F]**1** in control and LPS mice; (**C**) regional brain TACs of [^18^F]**1** in control and LPS mice.

Dynamic PET/CT images of control and LPS mice were acquired, and the brain uptake was higher in the LPS mouse than in the control mouse (Figure 4A). Whole brain time activity curve (TAC) analysis demonstrated that the uptakes peaked within 3 min after radioligand injection with a gradual decrease over time (Figure 4B). Regional brain TAC analysis revealed relatively heterogeneous distribution. In the control mouse, there was uptake in the decreasing order of the thalamus, striatum, hypothalamus, frontal cortex, and cerebellum (Figure 4C). In the 5.5-week-old mice used in our study, the frontal cortex and cerebellum had similar levels of uptake, although the cerebral cortex has markedly higher CSF1R expression than does the cerebellum in older adult mice [37]. In the LPS mouse, a pattern similar to that of the control mouse was observed, with high uptake in the thalamus and striatum, and intermediate to low uptake in the hypothalamus, cerebellum, and frontal cortex (Figure 4C). This result showed that regional brain uptake was enhanced in the LPS mouse over the control mouse.

Due to the relatively low molar activity of [^18^F]**1** (32–38 GBq/μmol) compared with that of [^11^C]CPPC (122.9 ± 25.8 GBq/μmol and 977 ± 451 GBq/μmol) reported in previous studies [18,26], we cannot exclude the possibility that the CSF1R might have been partly blocked by the non-radioactive ligand present in the final product, particularly in the control mouse brain where the receptor density was low (Figure 3). In the control mouse brain, therefore, the uptake of [^18^F]**1** might have been lower than that would have been achieved using the radioligand with higher molar activity. However, this preliminary result provided information on the uptake patterns of the new radioligand [^18^F]**1** in control and LPS mouse brains.

**Figure 5:**
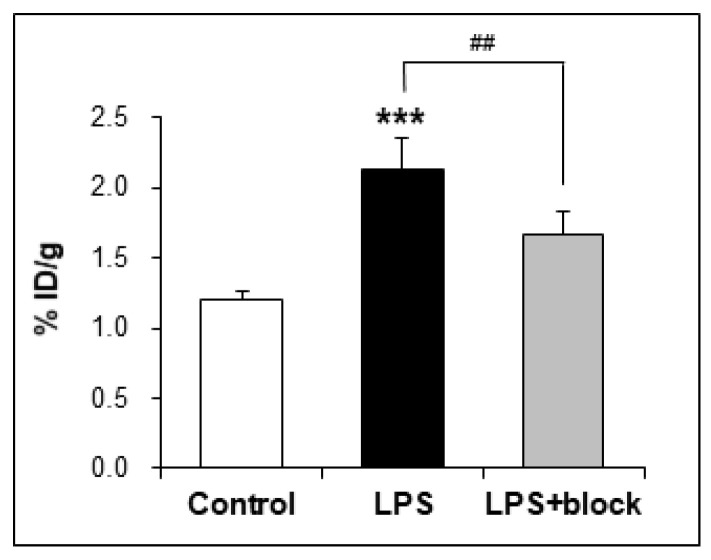
Ex vivo biodistribution result obtained 45 min after i.v. injection of [^18^F]**1** into mice: brain uptakes of control mice (white), LPS mice (black), and LPS mice pretreated with CPPC (LPS+block) (grey). Data are expressed as mean % ID/g ± S.D. (n = 4). *** *p* < 0.001 vs. control. ^##^ *p* < 0.01 vs. LPS mice.

Ex vivo biodistribution studies were performed in three groups of mice (control, LPS, and LPS+block groups) at 45 min after intravenous (i.v.) injection of [^18^F]**1**. In the blocking group (LPS+block), LPS-treated mice were injected i.p. with CPPC (1 mg/kg) at 5 min prior to i.v. injection of the radioligand. Dynamic PET/CT studies of control and LPS mice showed minimal changes in brain uptake between 40 and 60 min after radioligand injection, and [^11^C]CPPC uptake was compared among the three mice groups at 45 min after radioligand injection [18]. Therefore, we performed the ex vivo biodistribution studies at 45 min after injection of [^18^F]**1** (Table 2). Whole brain uptake was 1.20 ± 0.06% ID/g in control mice, whereas the uptake was elevated to 2.14 ± 0.19% ID/g in LPS mice, a 78% increase (Table 2, Figure 5). In the blocking study in LPS mice, the brain uptake decreased to 1.67 ± 0.16% ID/g from 2.14 ± 0.19% ID/g in the presence of CPPC (1 mg/kg), a 22% inhibition (Table 2, Figure 5). The relatively low level of inhibition can be attributed to the injection route and time of the blocking agent, i.p. administration 5 min prior to radioligand injection, which might not have allowed time for sufficient blocking. However, most of the PET radioligands currently developed for CSF1R imaging have low uptake or high non-specific binding in the brains of rodents and non-human primates [25,27,28]. Moreover, a recent in vitro study of [^3^H]CPPC showed off-target binding of CPPC to quite a number of kinases in the CNS; thus, [^11^C]CPPC may have low selectivity for CSF1R [38]. In this context, further blocking studies of [^18^F]**1** need to be conducted using the optimized injection time points of the blocking agents and using other potent CSF1R inhibitors as the blocking agents. High radioactivity accumulated in the liver of both control and LPS mice (6.87 ± 0.48% ID/g and 6.04 ± 0.91% ID/g, respectively), indicating that the radioligand was excreted through the hepatobiliary system (Table 2). It is also interesting to note that the liver uptake in the LPS+block mice was inhibited by 48% in the presence of CPPC, despite the similar uptakes in control and LPS mice (Table 2). This may be due to the blocking effect by an excess amount of CPPC taken up by the liver for metabolism and subsequent excretion. Low levels of bone uptake suggested that the radioligand did not suffer from in vivo metabolic defluorination. This result was consistent with those of other 4-(2-[^18^F]fluoroethyl)piperazinyl compounds [39,40,41]. In addition, these biodistribution data matched with the whole-body PET images of the control and LPS mice (Figure S2).

**Figure 6:**
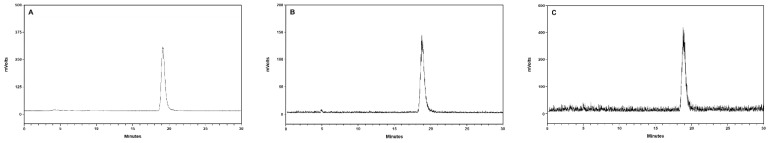
HPLC chromatograms of the brain homogenates obtained at 5 and 30 min after injection of [^18^F]**1** into control mice. (**A**) [^18^F]**1**; (**B**) brain homogenates at 5 min; (**C**) brain homogenates at 30 min.

In order to confirm that the brain uptake was due to the radioligand, we analyzed the brain homogenates and blood samples of control mice using HPLC. The radioligand was intact in the brain for the duration of the metabolism study (Figure 6), suggesting that the radioligand underwent little metabolism in the brain. However, approximately 65% of the radioligand in the blood samples was converted to polar radiometabolites within 30 min after injection (Figure S3). Therefore, this result implied that the brain uptake in mice was due to the radioligand but not the radiometabolites.

Although [^11^C]CPPC was reported as a lead PET radioligand for CSF1R imaging [18], the recent study revealed that CPPC has off-target binding in the CNS [38]. Radioligand [^18^F]**1** was designed based on CPPC, and therefore, further studies are warranted to confirm whether [^18^F]**1** has selective binding to CSF1R over other targets, including kinases in the brain.

## 3. Materials and Methods

### 3.1. General

Chemicals were purchased from Merck (Darmstadt, Germany), Tokyo Chemical Industry (Tokyo, Japan), and Xian Feidian Chemical (Féngchéng Shi, China). CPPC was purchased from ChemScene (Shanghai, China) and LPS (strain O111:B4 from Calbiochem) was obtained from Merck. NMR (^1^H, ^13^C, and ^19^F) spectra were obtained using Bruker AvanceIII-600 (600 MHz) and Avance 500 (500 MHz) spectrometers (Rheinstetten, Germany). Chemical shifts (δ) were reported as the ppm downfield of tetramethylsilane. Electron ionization (EI) mass spectra were obtained using a JMS-700 MStation instrument (JEOL, Tokyo, Japan). Ligand **1** used for biological studies is >95% pure by HPLC analysis (Supplementary Materials).

[^18^F]Fluoride was produced by the ^18^O(p,n)^18^F reaction using a PETtrace 880 cyclotron (GE Healthcare, Uppsala, Sweden). Kryptofix (K_2.2.2_) was purchased from ABX (Radeberg, Germany). TLC was performed on Merck F254 silica plates and analyzed using a Bioscan radio-TLC scanner (Eckert & Ziegler, Hopkinton, MA, USA). Purification and analysis of the radioligand were performed by HPLC (Thermo Fisher Scientific, Waltham, MA, USA) equipped with a semi-preparative column (YMC-Pack C18, 5 μm, 10 × 250 mm) and an analytical column (YMC-Pack C18, 5 μm, 4.6 × 250 mm), respectively. Molar activity of the radioligand was measured using HPLC (Agilent Technologies, Santa Clara, CA, USA) equipped with an analytical column (YMC-Pack C18, 5 μm, 4.6 × 250 mm). Eluates were monitored simultaneously using NaI(T1) radioactivity and UV (254 nm) detectors. Radioactivity was measured in a dose calibrator (Biodex Medical Systems, Shirley, NY, USA).

### 3.2. Synthesis of Ligand ***1***

#### 3.2.1. *tert*-Butyl 4-(4-Nitro-3-(piperidin-1-yl)phenyl)piperazine-1-carboxylate (**2**)

To a solution of 1-(5-chloro-2-nitrophenyl)piperidine (230 mg, 0.96 mmol) and *tert*-butyl piperazine-1-carboxylate (356 mg, 1.91 mmol) in dimethyl sulfoxide (DMSO) (3 mL) was added K_2_CO_3_ (396 mg, 2.87 mmol) under a nitrogen atmosphere. The reaction mixture was stirred at 110 °C for 16 h, after which it was extracted with CH_3_COOCH_2_CH_3_ (EtOAc) and water, and the organic layer was washed with brine and dried over Na_2_SO_4_. Flash column chromatography (5:1 hexane-EtOAc) gave **2** (315 mg, 84.1%) as a yellow solid. ^1^H NMR (500 MHz, CDCl_3_) δ 8.02 (d, *J* = 9.5 Hz, 1H), 6.43 (d, *J* = 8.5 Hz, 2H), 3.60 (t, *J* = 5 Hz, 4H), 3.39 (s, br, 4H), 3.12 (s, br, 4H), 1.80 (s, br, 4H), 1.65 (s, br, 2H), 1.49 (s, 9H). MS (EI) *m*/*z* 390 (M^+^): HRMS calcd for C_20_H_30_N_4_O_4_, 390.2267; found, 390.2272.

#### 3.2.2. *tert*-Butyl 4-(4-Amino-3-(piperidin-1-yl)phenyl)piperazine-1-carboxylate (**3**)

A solution of **2** (175 mg, 0.45 mmol) and SnCl_2_·2H_2_O (506 mg, 2.25 mmol) in ethanol (1 mL) was stirred at 70 °C for 30 min under nitrogen. After cooling to room temperature, the reaction vial was cooled in an ice-water bath, and the pH was adjusted to slightly basic (pH 7–8) by the addition of saturated NaHCO_3_ solution (2–2.5 mL). The reaction mixture was extracted with EtOAc and water, and the organic layer was washed with brine and dried over Na_2_SO_4_. Flash column chromatography (20:1 CH_2_Cl_2_-CH_3_OH) gave **3** (135.8 mg, 83.8%) as a brown solid. ^1^H NMR (500 MHz, CDCl_3_) δ 6.74 (s, br, 1H), 6.69 (d, *J* = 8 Hz, 1H), 6.61 (s, br, 1H), 3.60 (s, br, 4H), 2.99 (s, br, 4H), 2.87 (s, br, 4H), 1.58 (s, br, 4H), 1.48 (s, 9H). MS (EI) *m*/*z* 360 (M^+^): HRMS calcd for C_20_H_32_N_4_O_2_, 360.2525; found, 390.2529.

#### 3.2.3. *tert*-Butyl 4-(4-(5-Cyanofuran-2-carboxamido)-3-(piperidin-1-yl)phenyl)piperazine-1-carboxylate (**4**)

To a solution of **3** (23.5 mg, 0.065 mmol), 5-cyanofuran-2-carboxylic acid (9.6 mg, 0.07 mmol), and HATU (26.5 mg, 0.07 mmol) in dimethylformamide (DMF) (0.4 mL), DIPEA (22.6 μL, 0.13 mmol) was added under a nitrogen atmosphere. The reaction mixture was stirred at room temperature for 16 h. At the end of reaction, the mixture was extracted with EtOAc and water, and the organic layer was washed with brine and dried over Na_2_SO_4_. Flash column chromatography (9:1 CH_2_Cl_2_-CH_3_OH) gave **4** (26.8 mg, 86.2%) as a yellow solid. ^1^H NMR (500 MHz, CDCl_3_) δ 9.53 (s, 1H), 8.34 (d, *J* = 8 Hz, 1H), 7.26 (d, *J* = 4 Hz, 1H), 7.22 (d, *J* = 3.5 Hz, 1H), 6.80 (s, 1H), 6.73 (s, br, 1H), 3.60 (s, br, 4H), 3.12 (s, br, 4H), 2.85 (s, br, 4H), 1.81 (s, br, 4H), 1.65 (s, br, 2H), 1.49 (s, 9H). MS (EI) *m*/*z* 479 (M^+^): HRMS calcd for C_26_H_33_N_5_O_4_, 479.2533; found, 479.2535.

#### 3.2.4. 5-Cyano-*N*-(4-(piperazin-1-yl)-2-(piperidin-1-yl)phenyl)furan-2-carboxamide (**5**)

To a solution of **4** (20.1 mg, 0.042 mmol) in CH_2_Cl_2_ (200 μL) was added CF_3_COOH (16 μL, 0.21 mmol) at 0 °C, and the reaction mixture was stirred at room temperature for 16 h. At the end of reaction, the mixture was concentrated under reduced pressure. Flash column chromatography (9:1 CH_2_Cl_2_-CH_3_OH) gave **5** (14.9 mg, 93.4%) as a yellow solid. ^1^H NMR (500 MHz, CDCl_3_) δ 9.52 (s, 1H), 8.37 (d, *J* = 9 Hz, 1H), 7.27 (d, *J* = 3.5 Hz, 1H), 7.23 (d, *J* = 3.5 Hz, 1H), 6.79 (d, *J* = 2.5 Hz, 1H), 6.75 (dd, *J* = 9 and 2.5 Hz, 1H), 3.41 (d, *J* = 6 Hz, 4H), 3.38 (d, *J* = 6 Hz, 4H), 2.85 (t, *J* = 4.5 Hz, 4H), 1.84–1.79 (m, 4H), 1.58 (s, br, 2H). MS (EI) *m*/*z* 379 (M^+^): HRMS calcd for C_21_H_25_N_5_O_2_, 379.2008; found, 379.2009.

#### 3.2.5. 5-Cyano-*N*-(4-(4-(2-fluoroethyl)piperazin-1-yl)-2-(piperidin-1-yl)phenyl)furan-2-carboxamide (**1**)

A mixture of **5** (19.7 mg, 0.052 mmol), 1-fluoro-2-iodoethane (13.9 mg, 0.08 mmol), and K_2_CO_3_ (21.8 mg, 0.158 mmol) in DMF was stirred at 65 °C for 6 h under nitrogen. At the end of the reaction, the mixture was extracted with CH_2_Cl_2_ and water, and the organic layer was washed with water and dried over Na_2_SO_4_. Flash column chromatography (1:1 hexane-EtOAc) gave **1** (8.2 mg, 36.2%) as a yellow solid. ^1^H NMR (600 MHz, CDCl_3_) δ 9.53 (s, 1H), 8.32 (d, *J* = 8.9 Hz, 1H), 7.25 (d, *J* = 3.7 Hz, 1H), 7.22 (d, *J* = 3.7 Hz, 1H), 6.80 (s, 1H), 6.73 (d, *J* = 8.9 Hz, 1H), 4.68 (dt, *J* = 47.6 and 4.6 Hz, 2H), 3.22 (t, *J* = 4.4 Hz, 4H), 2.84–2.81 (m, 6H), 2.76–2.73 (m, 6H), 1.82–1.79 (m, 4H). ^13^C NMR (151 MHz, CDCl_3_) δ 153.2, 152.3, 148.6, 144.1, 126.5, 125.3, 123.6, 120.0, 114.6, 112.3, 110.6, 109.3, 82.5 (d, *J* = 169.0 Hz), 58.2 (d, *J* = 19.6 Hz), 53.9, 49.4, 30.3, 27.4, 24.1. ^19^F NMR (282 MHz, CDCl_3_) δ -219.95. MS (EI) *m*/*z* 425 (M^+^): HRMS calcd for C_23_H_28_FN_5_O_2_, 425.2227; found, 425.2223.

### 3.3. Synthesis of Precursor *(**7**)*

#### 3.3.1. 5-Cyano-*N*-(4-(4-(2-hydroxyethyl)piperazin-1-yl)-2-(piperidin-1-yl)phenyl)furan-2-carboxamide (**6**)

To a solution of compound **5** (50 mg, 0.13 mmol) and 1-bromo-2-hydroxyethane (14 μL, 0.2 mmol) in DMF, K_2_CO_3_ (27.3 mg, 0.2 mmol) was added. The reaction mixture was stirred at room temperature for 16 h under nitrogen. After the reaction was quenched with saturated NH_4_Cl solution, the mixture was extracted with CH_2_Cl_2_ and water, and the organic layer was washed with water and dried over Na_2_SO_4_. Flash column chromatography (1:1 hexane-EtOAc) gave **6** (25.2 mg, 45.8%) as a yellow solid. ^1^H NMR (500 MHz, CDCl_3_) δ 9.53 (s, 1H), 8.34 (d, *J* = 9 Hz, 1H), 7.26 (d, *J* = 5 Hz, 1H), 7.22 (d, *J* = 3.5 Hz, 1H), 6.79 (s, 1H), 6.74 (d, *J* = 9 Hz, 1H), 3.77 (m, 2H), 3.31 (m, 4H), 2.85–2.83 (m, 8H), 2.77 (s, 2H), 1.83–1.79 (m, 4H), 1.66 (s, 3H). MS (EI) *m*/*z* 423 (M^+^).

#### 3.3.2. 5-Cyano-*N*-(4-(4-(2-chloroethyl)piperazin-1-yl)-2-(piperidin-1-yl)phenyl)furan-2-carboxamide (**7**)

To compound **6** (10 mg, 0.024 mmol) in CH_2_Cl_2_ was added *p*-toluenesulfonyl chloride (13.7 mg, 0.072 mmol). After the addition of triethylamine (10 μL, 0.072 mmol) and 4-(dimethylamino)pyridine (2.4 mg, 0.02 mmol) at 0 °C, the reaction mixture was stirred at room temperature for 16 h. The reaction was then quenched with saturated NH_4_Cl solution, the reaction mixture was extracted with EtOAc and water, and the organic layer was washed with water and dried over Na_2_SO_4_. Flash column chromatography (20:1 CH_2_Cl_2_-CH_3_OH) gave **7** (9 mg, 67.8%) as a yellow solid. ^1^H NMR (600 MHz, CDCl_3_) δ 9.53 (s, 1H), 8.32 (d, *J* = 8.8 Hz, 1H), 7.25 (d, *J* = 3.7 Hz, 1H), 7.22 (d, *J* = 3.7 Hz, 1H), 6.79 (s, 1H), 6.72 (d, *J* = 8.9 Hz, 1H), 3.65 (t, *J* = 6.6 Hz, 2H), 3.2 (s, 4H), 2.84–2.70 (m, 12H), 1.82–1.78 (m, 4H). ^13^C NMR (151 MHz, CDCl_3_) δ 153.2, 152.3, 148.5, 144.1, 128.0, 126.5, 125.3, 123.6, 120.0, 114.6, 112.4, 110.6, 109.4, 59.7, 53.9, 49.5, 26.9, 24.1. MS (EI) *m*/*z* 441 (M^+^), 443 (M^+^ + 2): HRMS calcd for C_23_H_28_ClN_5_O_2_, 441.1932; found, 441.1933 (M^+^), 443.1916 (M^+^ + 2).

### 3.4. Radiochemical Synthesis

[^18^F]Fluoride (≤1500 MBq) was placed in a Vacutainer containing K_2_CO_3_ (0.25 mg in 100 μL water) and K_2.2.2_ (1.5 mg in 200 μL acetonitrile). Three azeotropic distillations were performed using acetonitrile at 90 °C (oil bath) under a gentle stream of N_2_. The resulting K[^18^F]F-K_2.2.2_ complex was dissolved in DMSO (200 μL) and transferred to a reaction vial containing precursor **7** (1 mg, 2.26 μmol). This reaction solution was allowed to stir at 120 °C for 10 min. After the reaction mixture was cooled to room temperature, it was extracted with saturated NaHCO_3_ solution (aq.) and EtOAc. The organic layer was passed through a Na_2_SO_4_ plug, and then the solvent was dried under a gentle stream of N_2_ (90 °C). The residue was purified by HPLC using a semi-preparative column eluted with a 44:56 mixture of 0.05 M ammonium formate (aq.) and acetonitrile at a flow rate of 3 mL/min. The desired product eluted between 28 and 29 min was collected and dried under a gentle stream of N_2_ (90 °C). The product was re-dissolved in ethanol and diluted with saline to give a final solution of 10% ethanol in saline.

Radiochemical and chemical purities of the product were measured using HPLC. These measurements were performed using an analytical column eluted with a 20:80 mixture of 0.05 M ammonium formate (aq.) and acetonitrile at a flow rate of 1 mL/min. Molar activity was determined by comparing the UV peak area of the desired radioactive peak and the UV peak areas of different concentrations of non-radioactive ligand on HPLC. This was performed using an analytical column under the same conditions described above. The identity of [^18^F]**1** was confirmed by co-injecting the radioligand and the corresponding non-radioactive ligand into the HPLC system.

### 3.5. In Vitro Studies

#### 3.5.1. Binding Assay

The binding affinities of CPPC and ligand **1** for CSF1R were measured using a commercially available assay kit (Thermo Fisher Scientific, Z’-LYTE kinase assay kit-tyrosine 1 peptide) and CSF1R (Abcam, Cambridge, UK). CPPC or **1** was dissolved in DMSO and serially diluted and then added to the reaction buffer that contained 50 mM HEPES, 0.01% BRIJ-35, 10 mM MgCl_2_, 1 mM EGTA, 1 mM ATP, and CSF1R (1 ng). The final concentration of DMSO in the incubation mixture was 1%. The mixtures were incubated at room temperature for 1 h, followed by development reaction incubations at room temperature for 1 h. The fluorescence intensity of the incubation mixtures was measured (excitation, 400 nm; emission, 445 nm (donor) and 520 nm (acceptor)) using a Mithras^2^ LB 943 monochromator multimode microplate reader (Berthold Technologies, Bad Wildbad, Germany), and the phosphorylation extent of the FRET-peptide was calculated. All experiments were performed in triplicate, and the IC_50_ values represent the average of three experimental determinations.

#### 3.5.2. Partition Coefficient

Radioligand [^18^F]**1** was dissolved in 800 μL 1-octanol and mixed with 800 μL water, vortexed vigorously for 5 min, and centrifuged. Two layers were separated, and 100 μL aliquots of the 1-octanol and aqueous layers were removed and counted. Samples from the 1-octanol and aqueous layers were repartitioned until consistent values were obtained. The log *P* is expressed as the logarithm of the ratio of the counts per minute from octanol versus that of water.

#### 3.5.3. Binding Study of [^18^F]**1** to CSF1R

A binding study of [^18^F]**1** for CSF1R was performed using detachable 96-well polystyrene plates (Nest Scientific, Rahway, NJ, USA). The plates were coated with 50 μL of CSF1R (200 μg/mL) in 0.05 M carbonate-bicarbonate buffer (pH 9.6). Non-coated plates were used for a control experiment. The CSF1R-coated and non-coated plates were incubated at 4 °C overnight and washed three times with PBS-T (20 mM PBS (pH 7.4) with 0.05% Tween-20). The unoccupied sites were blocked with blocking buffer (5% BSA in PBS-T) at room temperature for 1 h, and the plates were washed three times with PBS-T. [^18^F]**1** was dissolved in DMSO, and two-fold serial dilutions ranging from 1:100 to 1:400 were made from the radioligand solution. The diluted radioligand solutions were added to the CSF1R-coated and non-coated wells, which were then incubated at room temperature for 1 h. After incubation, the plates were washed three times with PBS-T. The wells were detached, and the radioligand bound to the wells was measured using a gamma counter and expressed as counts per minute (cpm). All experiments were performed in triplicate.

### 3.6. In Vivo Studies

#### 3.6.1. Animals

ICR male mice aged between 5 and 6 weeks were used in this study. The mice were provided drinking water and a normal diet ad libitum and were maintained under a 12 h light-dark cycle at 24 ± 1 °C with 50% humidity. The LPS-treated mouse model was prepared by injecting LPS (10 mg/kg in saline) i.p. into mice, and all experiments were carried out on the third day after LPS injection. Tissue radioactivity was counted using an automatic gamma counter (PerkinElmer, Waltham, MA, USA). MicroPET/CT images were acquired using an Inveon microPET/CT scanner (Siemens Medical Solutions, Knoxville, TN, USA).

All animal experiments were performed in accordance with the National Institutes of Health Guide for the Care and Use of Laboratory Animals and were approved by the Institutional Animal Care and Use Committee of Samsung Medical Center (20210104001).

#### 3.6.2. CSF1R Levels in Mouse Brain

Brain tissues were obtained from mice sacrificed by cervical dislocation on the third day after LPS (n = 3) or saline (control) treatment (n = 3) and lysed with 1 g/10 mL (*w*/*v*) tissue extraction buffer (Merck). To minimize variation, all samples were extracted at 4 °C or on ice. The CSF1R level in the brain tissues was determined by using a mouse CSF1R ELISA kit (Abcam) followed by absorbance measurement at 450 nm using an ELISA microplate reader (Molecular Devices, Sunnyvale, CA, USA).

#### 3.6.3. MicroPET/CT Imaging

Dynamic PET scans of mice (one control and one LPS) were initiated simultaneously with tail vein injection of [^18^F]**1** (7.4 ± 0.1 MBq/mouse) and performed for 60 min (15 s × 8 frames, 30 s × 10 frames, 60 s × 3 frames, 240 s × 5 frames, 300 s × 6 frames, 32 frames in total); CT images followed for 20 min. The PET images obtained were reconstructed using three-dimensional ordered subset expectation maximization and were processed using Inveon Research Workplace version 4.2 (Siemens Medical Solutions).

Analysis of dynamic PET data was performed using the PMOD 3.308 software (PMOD Technologies, Zurich, Switzerland). The CT image of the brain was fused manually with the mouse brain magnetic resonance image template (M. Mirrione), and then the PET image was applied for registration to the brain template with the transformation information of the CT image. Several volumes of interest (VOIs) were determined on the mouse brain template: whole brain, striatum (mean value of the left and right striata), thalamus, hypothalamus, frontal cortex, and cerebellum. From the results, whole brain and regional TACs were generated and presented as standardized uptake values (SUVs).

#### 3.6.4. Ex Vivo Biodistribution

ICR mice were divided into three groups: control, LPS, and LPS+block groups (n = 4 per group). In the LPS+block group, LPS-treated mice were injected i.p. with 100 μL of CPPC (1 mg/kg, 10% DMSO-saline) at 5 min prior to i.v. injection of the radioligand (910 ±47 kBq) dissolved in 10% ethanol in saline. Control and LPS-treated mice were injected i.p. with 100 μL of vehicle (10% DMSO-saline) at 5 min prior to the radioligand injection. At 45 min after radioligand injection, mice were sacrificed by cervical dislocation, and the blood and major tissues (brain, muscle, femur, liver, spleen, and kidney) were separated, weighed, and counted. Data are expressed as a percentage of injected dose per gram of tissue (% ID/g).

#### 3.6.5. Metabolism

ICR mice (n = 1 per time point) were injected with [^18^F]**1** (18.5 MBq) via a tail vein. At 5 and 30 min after injection, mice were sacrificed, and samples of brain and blood were obtained. The samples were homogenized in 1 mL of acetonitrile and centrifuged. The supernatants were analyzed by HPLC using an analytical column eluted with a 45:55 mixture of 0.05 M ammonium formate (aq.) and acetonitrile at a flow rate of 1 mL/min. Eluates were monitored using a NaI(T1) radioactivity detector.

### 3.7. Statistical Analysis

Data were analyzed using unpaired, two-tailed Student’s *t*-test using GraphPad Prism version 7.0 software, and differences at the 95% confidence level (*p* < 0.05) were considered significant.

## 4. Conclusions

In this study, we developed a ^18^F-labeled ligand for PET imaging of CSF1R. In vitro and in vivo evaluation of [^18^F]**1** conducted in this study suggested that the radioligand has high and specific binding to CSF1R. Further studies are warranted to investigate the selectivity of [^18^F]**1** for use in PET imaging of CSF1R.

## Figures and Tables

**Table 1:** IC_50_ values of CPPC and **1** for CSF1R.

Ligand	IC_50_ (nM)
CPPC	1.56 ± 0.08
**1**	3.42 ± 0.33

IC_50_ values are given as the mean ± S.D. of three measurements.

**Table 2:** Ex vivo biodistribution of [^18^F]**1** in control mice, LPS mice, and LPS mice pretreated with CPPC (LPS+block).

	Control	LPS	LPS+block
blood	0.93 ± 0.05	1.21 ± 0.11	1.30 ± 0.11
brain	1.20 ± 0.06	2.14 ± 0.19	1.67 ± 0.16
muscle	0.96 ± 0.10	1.03 ± 0.07	1.20 ± 0.19
femur	1.80 ± 0.09	1.95 ± 0.44	2.11 ± 0.37
liver	6.87 ± 0.48	6.04 ± 0.91	3.15 ± 0.62
spleen	1.58 ± 0.20	2.47 ± 0.19	1.45 ± 0.13
kidney	1.97 ± 0.05	2.23 ± 0.08	2.04 ± 0.10

Values (% ID/g) are given as the mean ± S.D. of groups, n = 4 mice.

## Data Availability

Data are available in the article and in the Supplementary Materials.

## Supplementary Materials

The following supporting information can be downloaded at https://www.mdpi.com/article/10.3390/ph15030276/s1. Analysis data of ligand **1** (NMR and HPLC) and radioligand (HPLC); Figure S1: IC_50_ curves; Figure S2: Whole-body PET images of mice; Figure S3: HPLC chromatograms of mouse blood samples.
