# Supplementary material for: Synthesis and Evaluation of a 18F-Labeled Ligand for PET Imaging of Colony-Stimulating Factor 1 Receptor
Source: Pharmaceuticals (Basel). 2022 Feb 23;15(3):276. doi: 10.3390/ph15030276 (PMC8954204; doi:10.3390/ph15030276)
Supplement: Supplementary file 1 [file pharmaceuticals-15-00276-s001.zip › pharmaceuticals-1559668-supplementary.pdf]

Supplementary Information

Synthesis and evaluation of a ^{18}F-labeled ligand for PET imaging of colony stimulating factor 1 receptor

Hyeokjin Lee [1,#], Ji-Hun Park [1,#], Hyunjung Kim [1], Sang-keun Woo [2], Joon Young Choi [1], Kyung-Han Lee[1,3] and Yearn Seong Choe [1,3,*]

[1]Department of Nuclear Medicine, Samsung Medical Center, Sungkyunkwan University School of Medicine, Seoul 06351, Korea
[2]Division of RI-convergence Research, Korea Institute of Radiological and Medical Sciences, Seoul 01812, Korea
[3]Department of Health Sciences and Technology, SAIHST, Sungkyunkwan University, Seoul 06355, Korea

Contents:

NMR (^{1}H, ^{13}C, and ^{19}F) spectra of ligand **1** 2
HPLC chromatogram of the radiofluorination reaction mixture 4
HPLC chromatogram of ligand **1** 5
HPLC chromatograms of radioligand [^{18}F]**1** 6
HPLC chromatograms of a mixture of [^{18}F]**1** and **1** 7
Figure S1. IC_{50} curves 8
Figure S2. Whole-body PET images of control and LPS mice 9
Figure S3. HPLC chromatograms of mouse blood samples 10

NMR spectra of ligand **1**

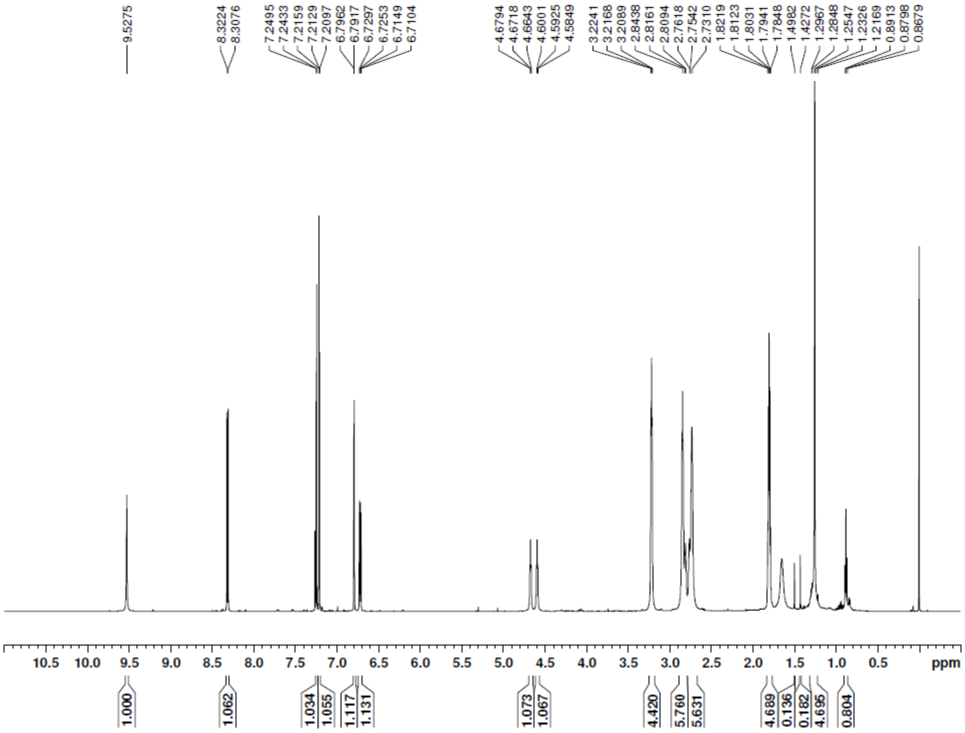

^{1}H NMR spectrum of **1**

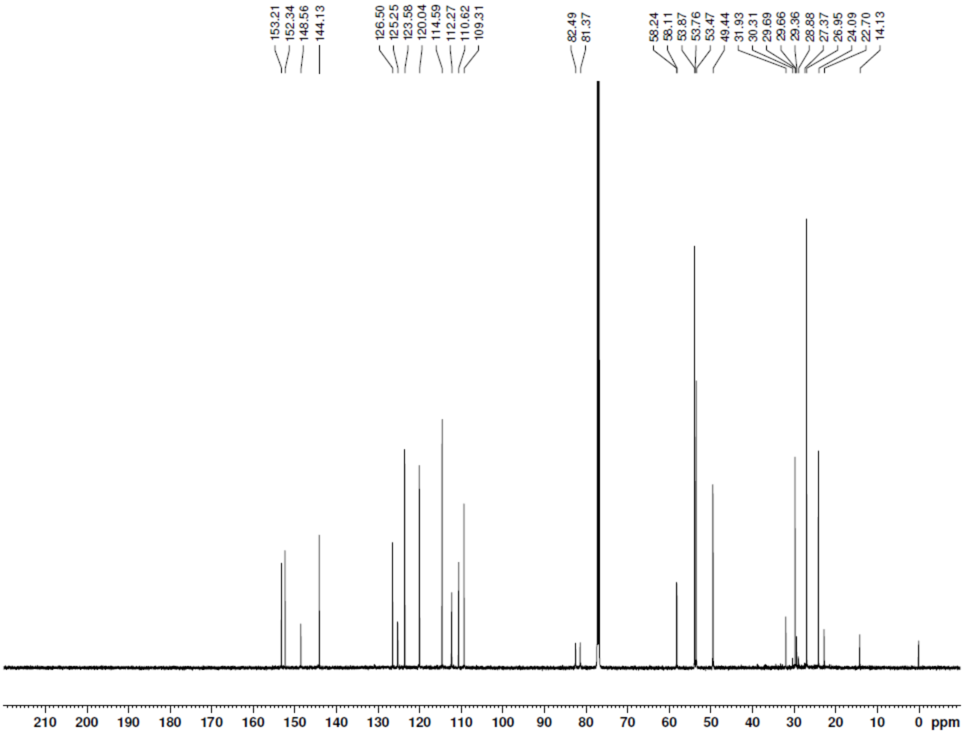

^{13}C NMR spectrum of **1**

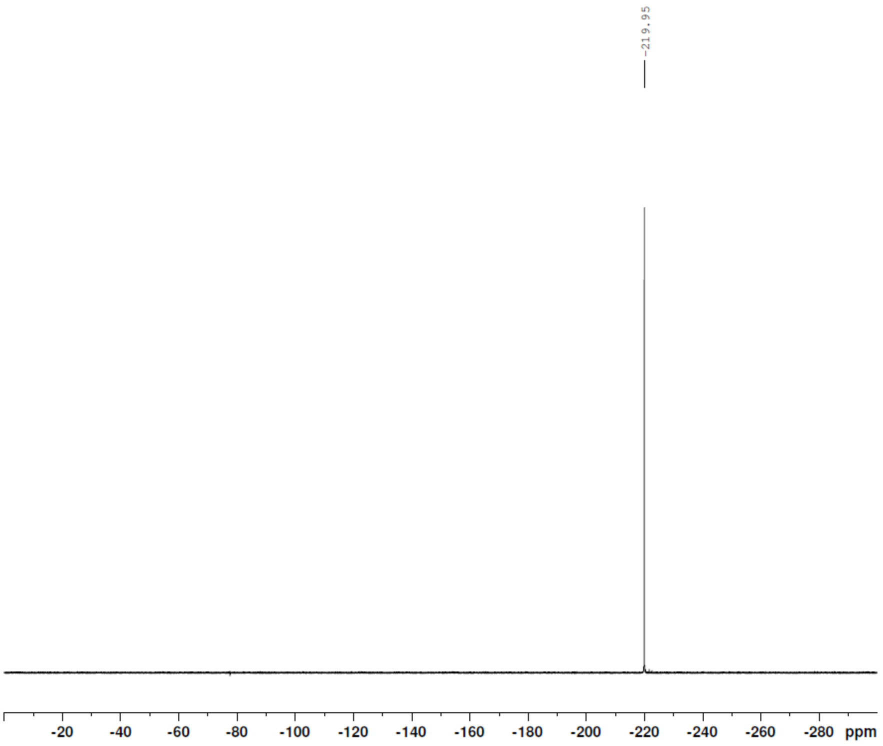

^{19}F NMR spectrum of **1**

HPLC chromatogram of ligand **1**

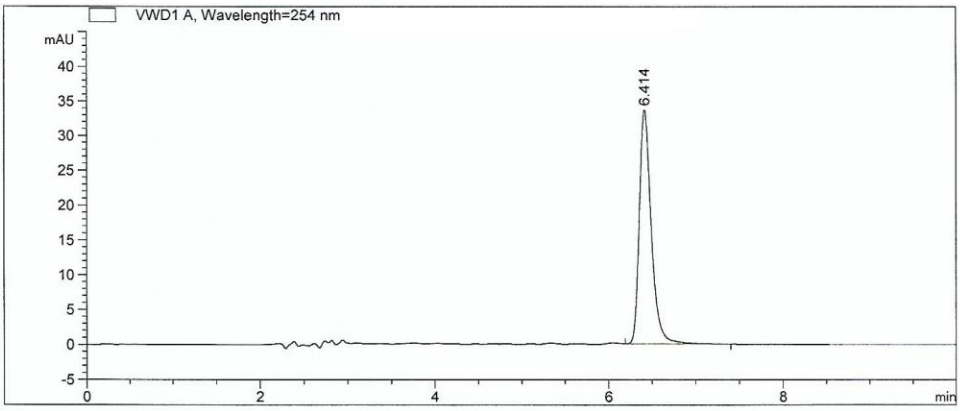

Area Percent Report

Sorted By	:	Signal
Multiplier	:	1.0000
Dilution	:	1.0000

Do not use Multiplier & Dilution Factor with ISTDs

Signal 1: VWD1 A, Wavelength=254 nm

Peak #	RetTime [min]	Type	Width [min]	Area [mAU*s]	Height [mAU]	Area %
1	6.414	BB	0.1440	320.47375	33.68629	100.0000
Totals :				320.47375	33.68629	

HPLC chromatogram of the radiofluorination reaction mixture

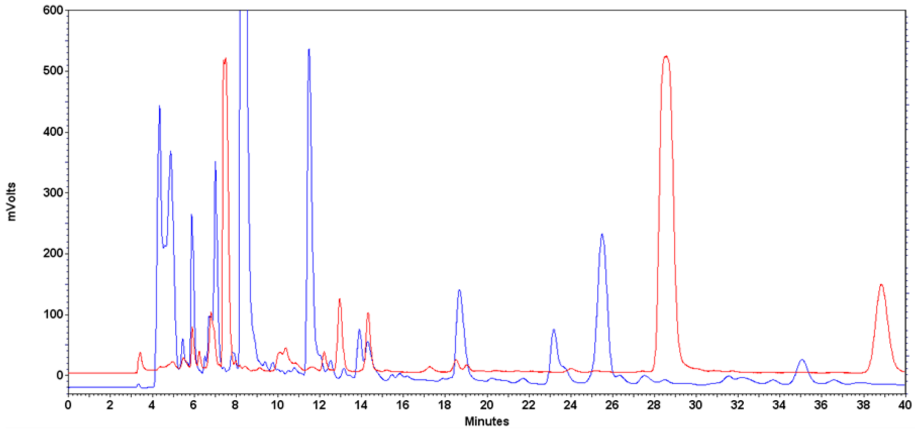

HPLC: Thermo Scientific
Semi-preparative HPLC column: YMC-Pack C18, 5 μ, 10 x 250 mm
HPLC solvents: 44:56 0.05 M ammonium formate–CH_3CN
Flow rate: 3 mL/min
Detection: Radioactivity (red) and UV (254 nm) (blue) detectors

HPLC chromatograms of radioligand [^{18}F]**1**

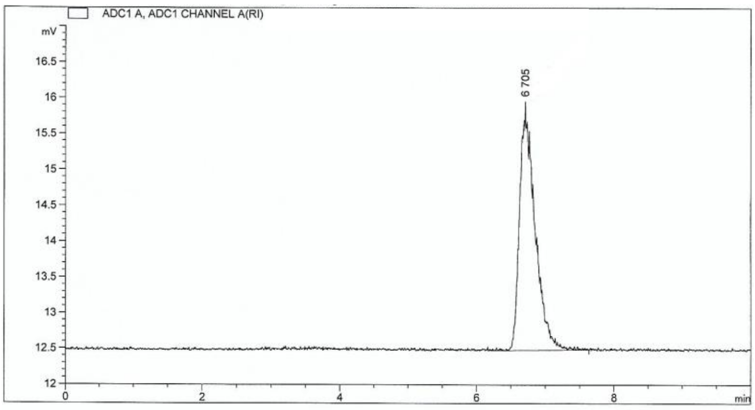

Area Percent Report

```
Sorted By             :      Signal
Multiplier            :      1.0000
Dilution              :      1.0000
Do not use Multiplier & Dilution Factor with ISTDs

Signal 1: ADC1 A, ADC1 CHANNEL A(RI)

Peak RetTime Type  Width     Area      Height     Area
  #   [min]        [min]   [mV*s]      [mV]        %
----|-------|----|-------|----------|----------|--------|
   1   6.705 MM    0.2560   53.81527    3.50360 100.0000

Totals :                    53.81527    3.50360
```

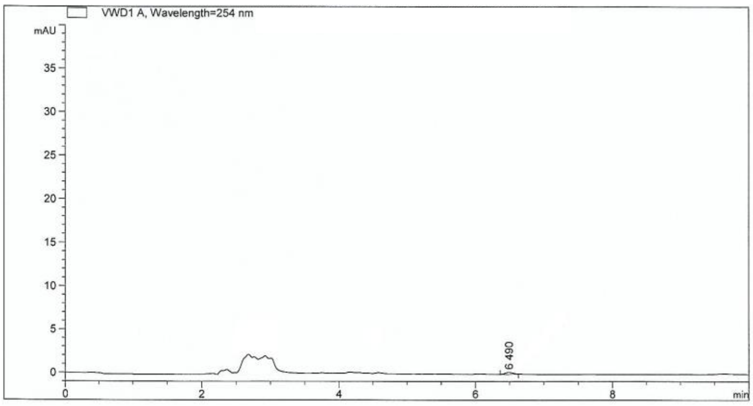

Area Percent Report

```
Sorted By             :      Signal
Multiplier            :      1.0000
Dilution              :      1.0000
Do not use Multiplier & Dilution Factor with ISTDs

Signal 1: VWD1 A, Wavelength=254 nm

Peak RetTime Type  Width     Area      Height     Area
  #   [min]        [min]   [mAU*s]     [mAU]       %
----|-------|----|-------|----------|----------|--------|
   1   6.490 MM    0.1451    1.95426 2.24549e-1 100.0000

Totals :                     1.95426 2.24549e-1
```

HPLC: Agilent Technologies
HPLC column: YMC-Pack C18, 5 µ, 4.6 x 250 mm
HPLC solvents: 20:80 0.05 M ammonium formate–CH_3CN
Flow rate: 1 mL/min
Detection: Radioactivity and UV (254 nm) detectors

HPLC chromatograms of a mixture of [^{18}F]**1** and **1**

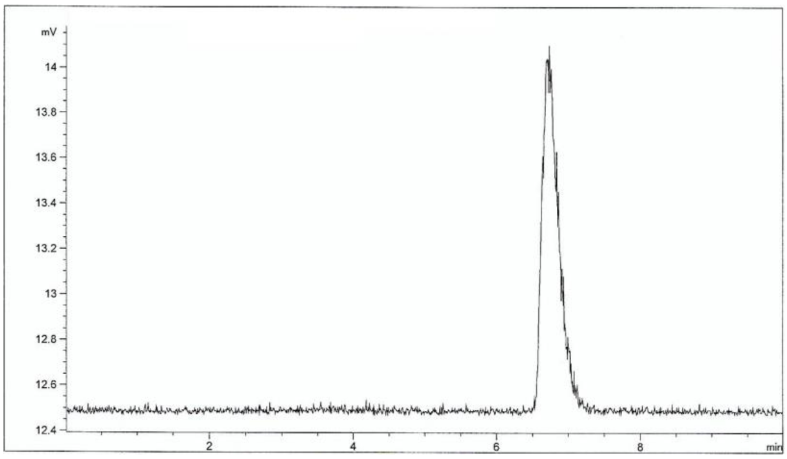

Radioactivity (t_R = 6.738 min)

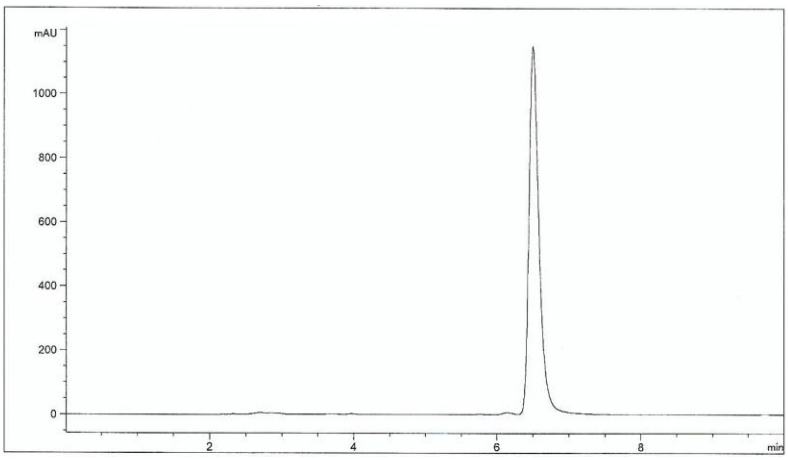

UV (t_R = 6.502 min)

HPLC: Agilent Technologies
HPLC column: YMC-Pack C18, 5 μ, 4.6 x 250 mm
HPLC solvents: 20:80 0.05 M ammonium formate–CH_3CN
Flow rate: 1 mL/min
Detection: Radioactivity and UV (254 nm) detectors

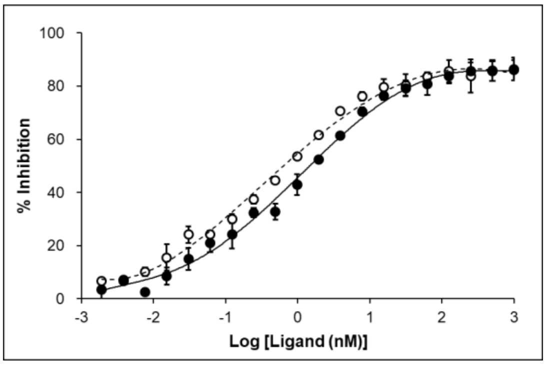

Figure S1. IC_{50} curves of CPPC (○) and **1** (●) for CSF1R

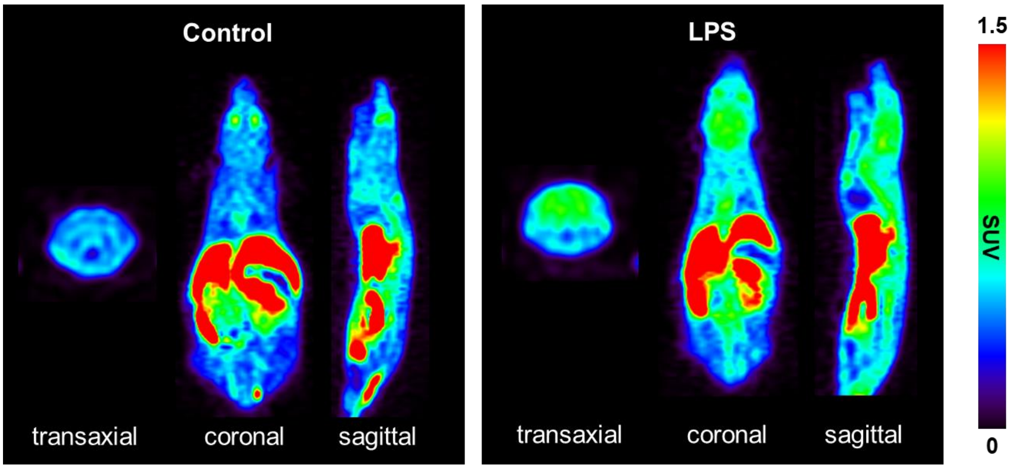

Figure S2. Whole-body PET images of control and LPS mice at 45 min after radioligand injection

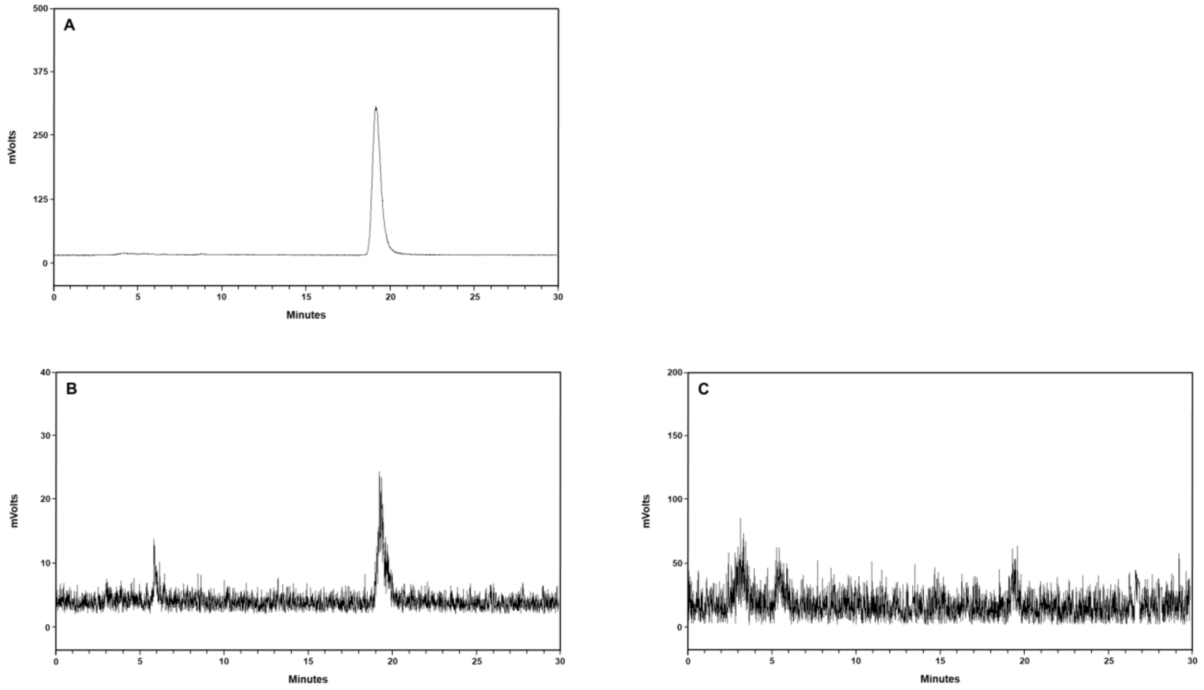

HPLC: Thermo Scientific
HPLC column: YMC-Pack C18, 5 µ, 4.6 x 250 mm
HPLC solvents: 45:55 0.05 M ammonium formate–CH_3CN
Flow rate: 1 mL/min
Detection: radioactivity detector

Figure S3. HPLC chromatograms of radioligand (A) and of the blood samples obtained at 5 min (B) and 30 min (C) after injection of [^{18}F]**1** into control mice
